# Fine-Tuning of Smad Protein Function by Poly(ADP-Ribose) Polymerases and Poly(ADP-Ribose) Glycohydrolase during Transforming Growth Factor β Signaling

**DOI:** 10.1371/journal.pone.0103651

**Published:** 2014-08-18

**Authors:** Markus Dahl, Varun Maturi, Peter Lönn, Panagiotis Papoutsoglou, Agata Zieba, Michael Vanlandewijck, Lars P. van der Heide, Yukihide Watanabe, Ola Söderberg, Michael O. Hottiger, Carl-Henrik Heldin, Aristidis Moustakas

**Affiliations:** 1 Ludwig Institute for Cancer Research, Science for Life Laboratory, Uppsala University, Uppsala, Sweden; 2 Department of Immunology, Genetics and Pathology, Science for Life Laboratory, Uppsala University, Uppsala, Sweden; 3 Institute of Veterinary Biochemistry and Molecular Biology, University of Zurich, Zurich, Switzerland; 4 Department of Medical Biochemistry and Microbiology, Science for Life Laboratory, Uppsala University, Uppsala, Sweden; Leiden University Medical Center, Netherlands

## Abstract

**Background:**

Initiation, amplitude, duration and termination of transforming growth factor β (TGFβ) signaling via Smad proteins is regulated by post-translational modifications, including phosphorylation, ubiquitination and acetylation. We previously reported that ADP-ribosylation of Smads by poly(ADP-ribose) polymerase 1 (PARP-1) negatively influences Smad-mediated transcription. PARP-1 is known to functionally interact with PARP-2 in the nucleus and the enzyme poly(ADP-ribose) glycohydrolase (PARG) can remove poly(ADP-ribose) chains from target proteins. Here we aimed at analyzing possible cooperation between PARP-1, PARP-2 and PARG in regulation of TGFβ signaling.

**Methods:**

A robust cell model of TGFβ signaling, i.e. human HaCaT keratinocytes, was used. Endogenous Smad3 ADP-ribosylation and protein complexes between Smads and PARPs were studied using proximity ligation assays and co-immunoprecipitation assays, which were complemented by in vitro ADP-ribosylation assays using recombinant proteins. Real-time RT-PCR analysis of mRNA levels and promoter-reporter assays provided quantitative analysis of gene expression in response to TGFβ stimulation and after genetic perturbations of PARP-1/-2 and PARG based on RNA interference.

**Results:**

TGFβ signaling rapidly induces nuclear ADP-ribosylation of Smad3 that coincides with a relative enhancement of nuclear complexes of Smads with PARP-1 and PARP-2. Inversely, PARG interacts with Smads and can de-ADP-ribosylate Smad3 *in vitro*. PARP-1 and PARP-2 also form complexes with each other, and Smads interact and activate auto-ADP-ribosylation of both PARP-1 and PARP-2. PARP-2, similar to PARP-1, negatively regulates specific TGFβ target genes (*fibronectin*, *Smad7*) and Smad transcriptional responses, and PARG positively regulates these genes. Accordingly, inhibition of TGFβ-mediated transcription caused by silencing endogenous PARG expression could be relieved after simultaneous depletion of PARP-1.

**Conclusion:**

Nuclear Smad function is negatively regulated by PARP-1 that is assisted by PARP-2 and positively regulated by PARG during the course of TGFβ signaling.

## Introduction

Signal transduction pathways, including transforming growth factor β (TGFβ), are controlled by negative regulatory mechanisms [1], [2], [3]. The TGFβ pathway is extensively studied due to its implication in early embryonic development, in specification of different organs, in homeostatic regulation of adult tissue integrity and due to its role in the development and progression of many diseases, including cardiovascular, fibrotic and malignant diseases [4], [5]. In the TGFβ pathway, negative regulation is exerted at multiple levels: at the level of the extracellular ligand and its access to the signaling receptors [6]; at the level of the type I and type II receptors that have serine/threonine kinase activity and phosphorylate intracellular Smad proteins or other signaling proteins [7]; at the level of the Smad proteins that form complexes with each other, e.g. the receptor-phosphorylated Smad2 and Smad3 (R-Smads) associate with Smad4 and together accumulate in the nucleus to regulate transcription [2]; and finally, at the level of many of the cytoplasmic and nuclear cofactors of the receptors and Smads, which are themselves regulated based on crosstalk with many other signaling pathways, and which provide the “context-dependent” function of the pathway [3], [8].

We recently established a mechanism of negative regulation of Smad activity taking place in the nucleus, based on the finding that Smad3 and Smad4 can associate with the nuclear ADP-ribosyl-transferase (ADP-ribosyltransferase diphtheria toxin-like 1, ARTD1), also known as poly(ADP-ribose) polymerase-1 (PARP-1) [9]. PARP-1 binds to Smad proteins and ADP-ribosylates them proximal to their DNA-binding domain, thus reducing their affinity to DNA and negatively regulating their transcriptional activity. A straightforward consequence of this biochemical modification is that PARP-1 negatively regulates gene responses to TGFβ signaling [9]. In a similar manner, PARP-1 suppresses the expression of TGFβ receptors in CD4-positive T cells and for this reason PARP-1 inhibitors enhance signaling by TGFβ [10]. In addition, PARP-1 can mediate positive gene responses to TGFβ as reported in studies of vascular smooth muscle cells [11]. A potential dual role of PARP-1 in mediating transcriptional responses is compatible with the current understanding of PARP-1 as a positive or negative regulator of transcription [12].

PARP-1 is the prototype of a large family of ADP-ribosyl-transferases (ARTDs) that enlists eighteen members acting towards diverse substrates in the nucleus, cytoplasm or mitochondria [12], [13], [14]. PARP-1 is best understood for its role in the DNA damage and repair response and the surveillance mechanisms that guarantee genomic integrity. Equally well established is the role of PARP-1 as a regulator of physiological transcription during embryonic development and adult tissue homeostasis [12], [13]. During transcription, PARP-1 builds poly(ADP)-ribose (PAR) chains on histones inside nucleosomes, affects the binding of histone H1 to nucleosomes, regulates DNA methylation, ADP-ribosylates the chromatin insulator protein CTCF and many DNA-binding transcription factors by modulating (usually negatively) their binding to DNA [12], [13]. In addition, PARP-1 and other PARP family members are known to auto-ADP-ribosylate as a mechanism that regulates their activity and residence to chromatin [15].

PARP-2 (ARTD2) is the second member of the family, it also localizes in the nucleus and shares a highly conserved catalytic domain with PARP-1 [14], however, it is a smaller protein, lacking many of the protein-protein interaction domains of PARP-1 and having a short N-terminal nuclear localization domain [16]. PARP-2 functions in a relatively similar manner with PARP-1 as both enzymes are intimately involved in the DNA-damage and repair response, chromatin remodeling and transcription and in the development of cancer [17]. During the DNA damage and nucleotide base excision-repair mechanisms PARP-2 functionally cooperates with PARP-1 by forming physical complexes with each other and affecting each other's catalytic activity [18]. In addition, PARP-2 can associate with the regulatory sequences of genes, such as *SIRT1*, an NAD-dependent deacetylase, repressing its expression and providing a mechanism that limits energy expenditure and mitochondrial function [19]. Interestingly, such transcriptional function of PARP-2 can be directly regulated by the histone acetyl-transferase P/CAF, which acetylates the N-terminal domain of PARP-2 and reduces the DNA-binding and auto-ADP-ribosylation activity of PARP-2 [20].

Protein ADP-ribosylation mediated by PARP-1 is dynamic and its turnover is controlled in part by the action of the enzyme poly(ADP-ribose) glycohydrolase (PARG) [21]. PARG can hydrolyze PAR chains, whereas mono(ADP-ribosyl) units are removed from target proteins by the action of the ADP-ribosyl hydrolase 3 (ARH3) and macrodomain-containing proteins such as MacroD1 [22]. A clear function of PARG is the regulation of chromatin remodeling during transcription as it antagonizes the functional effects of PARP-1 [23]. Genome-wide location analysis has demonstrated that both PARP-1 and PARG localize in distinct sets of gene regulatory sequences [24], [25]. Evidence based on comparative RNAi of PARP-1 versus PARG in breast cancer cells proposed that the two enzymes regulate gene expression in a coordinate and non-antagonistic manner, an intriguing finding that requires future mechanistic explanation [24].

In this investigation we analyzed the role of PARP-2 and PARG in association to PARP-1 during TGFβ signaling. Using proximity ligation assays (PLA) and immunoprecipitations, we demonstrate that TGFβ induces endogenous PARP-1/Smad3 and PARP-2/Smad2/3 complexes, while only having small effects on the PARP1/PARP-2 interaction. TGFβ also promotes endogenous Smad3 oligo(ADP-ribosyl)ation, while in vitro ADP-ribosylation experiments demonstrated that recombinant Smad3 or Smad4 could co-precipitate activated poly(ADP-ribosyl)ated PARP-1 and PARP-2. During TGFβ-regulated transcription, PARP-2 may act functionally in a similar manner as PARP-1, since PARP-2 suppressed TGFβ/Smad-dependent transcriptional responses. Finally, after demonstrating that PARG is capable of interacting with Smad proteins and de-ADP-ribosylating Smad3, we found that PARG is required for optimal transcriptional responses to TGFβ. Thus, in the case of TGFβ-mediated transcriptional regulation, PARP-2 complements PARP-1's negative regulation of nuclear Smad function, while PARG seems to antagonize PARP-1/2 and provide a balancing mechanism for the optimal control of signal-regulated transcription.

## Results

### Induction of ADP-ribosylation by TGFβ

**Figure 1:**
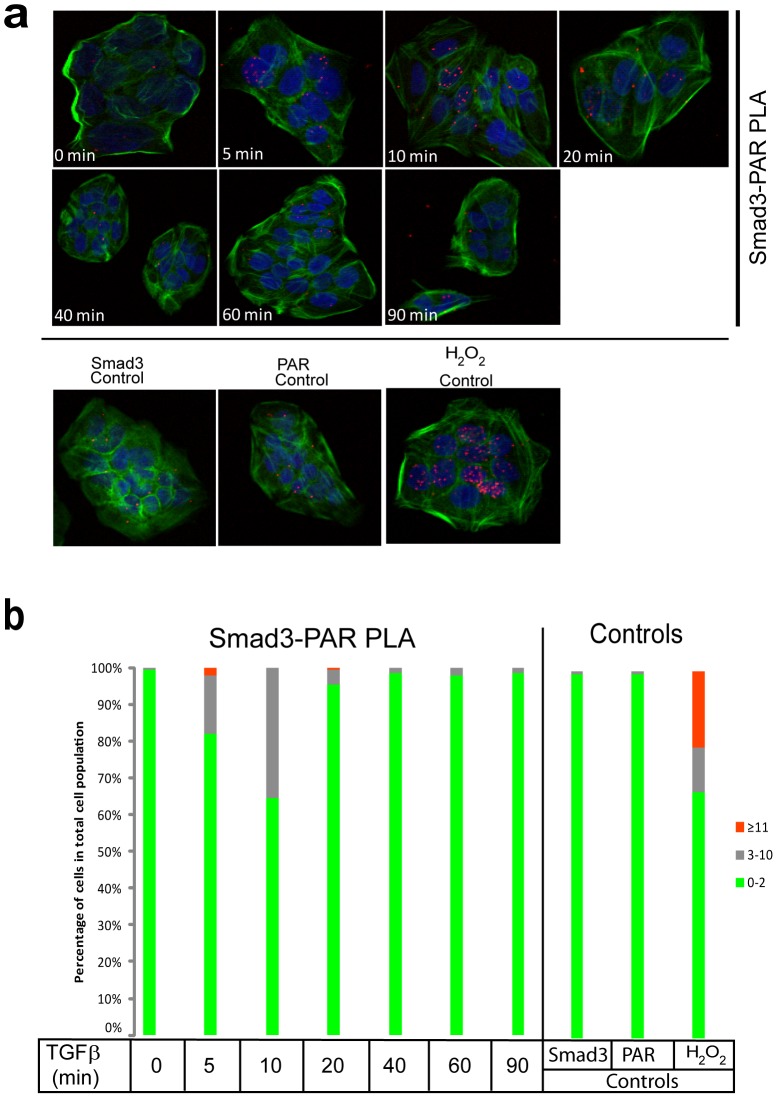
PLA of endogenous Smad3 ADP-ribosylation after TGFβ stimulation in HaCaT cells. (**a**) HaCaT cells were analyzed with PLA using antibodies against Smad3 and PAR chains after stimulation with vehicle (0 min) or with 2 ng/ml TGFβ1 for the indicated time periods. Specific RCA signals were detected in the nuclei. Cells stimulated with 10 mM hydrogen peroxide for 10 min served as positive control. PLA with single antibodies against Smad3 or PAR are shown as controls. PLA signals are shown in red, blue is DAPI staining for DNA and green is phalloidin staining for the actin cytoskeleton as a measure of overall cell architecture. (**b**) Quantification of the experiment shown in panel (a) using the DuolinkImageTool, with data plotted as a histogram divided in three classes according to the percent of cells that exhibit specific RCA signals: very low, 0–2 signals per cell [green]; low, 3–10 signals per cell [grey]; and high, >11 signals per cell [red]. The figure shows a representative experiment from three or more repeats.

We have previously provided evidence for the biochemical association of PARP-1 with Smad3 and Smad4, and for in vitro ADP-ribosylation of Smad3 and Smad4 [9]. In the present work we explored alternative techniques in order to demonstrate and quantify the extent of Smad protein ADP-ribosylation in living cells responding to TGFβ stimulation. We obtained reliable results when we applied in situ PLA [26], which provides a sensitive and quantitative method for detecting protein complexes or post-translational modifications of proteins. We focused mainly on Smad3, as this Smad associates stronger with PARP-1 and becomes ADP-ribosylated [9]. Using human immortalized keratinocytes (HaCaT) that are responsive to TGFβ signaling, we could observe rolling circle amplification (RCA) signals after applying antibodies against Smad3 and against PAR chains (Fig. 1). In the absence of TGFβ stimulation, very weak Smad3 ADP-ribosylation was detected that was indistinguishable from the negative controls of Smad3 or PAR antibody alone (Fig. 1). In contrast, TGFβ rapidly induced nuclear RCA signals that presumably represent ADP-ribosylation of Smad3 (Fig. 1). After quantification of the nuclear RCA signals using the DuolinkImageTool software, we could verify that nuclear ADP-ribosylation was induced at 5 min, was further enhanced at 10 min, already declined significantly at 20 min, and returned to steady but low levels up to 90 min after TGFβ stimulation (Fig. 1b), and the same low level persisted even up to 6 h after TGFβ stimulation (data not shown). Attempts to link the nuclear signals of Smad3-PAR to the activity of PARP-1 or PARP-2 using siRNA-mediated silencing of each protein failed for technical reasons, as PLA with the PAR antibody repeatedly failed when the cells were transfected (data not shown). As a positive control, we measured the endogenous Smad3 ADP-ribosylation after cell exposure to a rapid and acute dose of hydrogen peroxide (Fig. 1), which is known to induce strong PARP activity in the nucleus and can also induce stable Smad3-PARP-1 complexes [9]. Peroxide treatment in the absence of TGFβ stimulation caused dramatically higher levels of Smad3-PAR in the nuclei of HaCaT cells (Fig. 1). We conclude that PLA can reliably monitor endogenous Smad3 ADP-ribosylation in human cells in culture. This method allowed us for the first time to observe the rapid and relatively transient time course of Smad3 ADP-ribosylation in response to TGFβ signaling.

### TGFβ promotes protein complexes between Smads, PARP-1 and PARP-2

**Figure 2:**
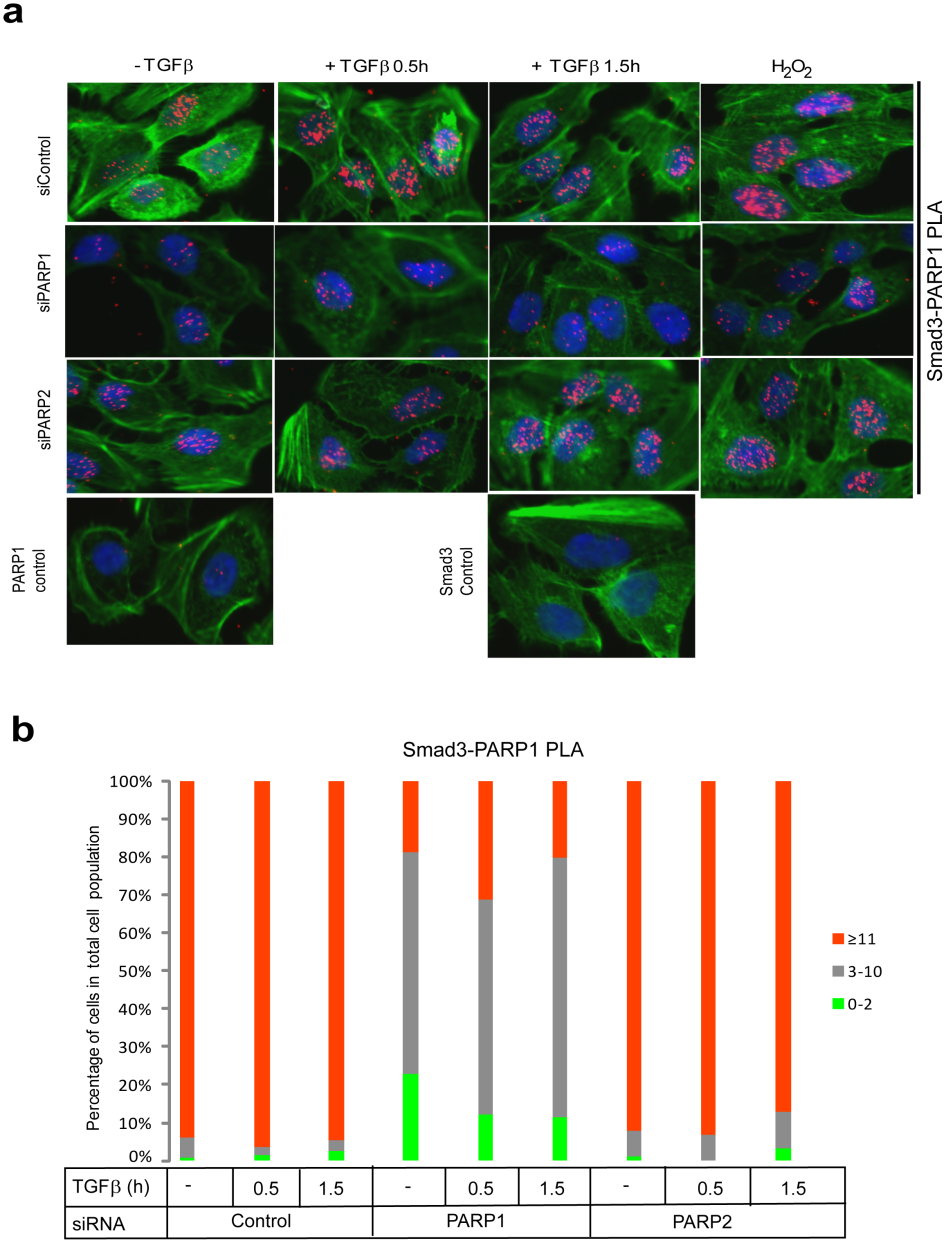
PLA of endogenous Smad3 and PARP-1 complexes in HaCaT cells. (**a**) HaCaT cells were analyzed with PLA using antibodies against Smad3 and PARP-1 after transfection with control or the indicated specific siRNAs and stimulation with vehicle (-TGFβ) or with 2 ng/ml TGFβ1 for the indicated time periods. Specific RCA signals were detected in the nuclei. Cells stimulated with 10 mM hydrogen peroxide for 10 min served as positive control. PLA with single antibodies against Smad3 or PARP-1 are shown as controls. PLA images are shown as in Fig. 1a. (**b**) Quantification of the experiment shown in panel (a) following the histogram method of Fig. 1b. The figure shows a representative experiment from three or more repeats.

We then analyzed endogenous complexes between Smad3 and PARP-1 using PLA, which also allowed us to simultaneously monitor the subcellular distribution of the complexes. We observed RCA signals derived from Smad3/PARP-1 protein complexes, exclusively in the nucleus (Fig. 2). After quantitation of the nuclear RCA signals we could verify that more than 95% of the cells in the epithelial monolayer exhibited detectable Smad3/PARP-1 complexes (Fig. 2b).

Smad3/PARP-1 complexes occurred even in the absence of TGFβ stimulation, but the incidence of complexes was higher after TGFβ stimulation for 0.5 h and lower after 1.5 h stimulation (Fig. 2b), which persisted even up to 6 h after TGFβ stimulation (data not shown). As a positive control, we measured the endogenous Smad3/PARP-1 complexes after exposure of cells to a rapid and acute dose of hydrogen peroxide, which led to a very dramatic accumulation of the nuclear RCA signals that was much stronger than the accumulation achieved by TGFβ (Fig. 2a). Multiple negative controls ascertained the specificity of detection of the endogenous Smad3/PARP-1 complexes: a) silencing of PARP-1 using siRNA reduced the nuclear RCA signals to almost background levels (Fig. 2). Similarly, silencing of PARP-1 significantly reduced the Smad3/PARP-1 complexes after cell treatment with peroxide (Fig. 2a). b) Silencing PARP-2 using siRNA only weakly reduced the observed Smad3/PARP-1 complexes, suggesting that PARP-2 is not essential for the formation of complexes between R-Smad and PARP-1 but contributes partially to the formation of the complexes (Fig. 2). c) Controls with single PARP-1 or Smad3 antibody gave the absolute background signal of this assay (Fig. 2a).

**Figure 3:**
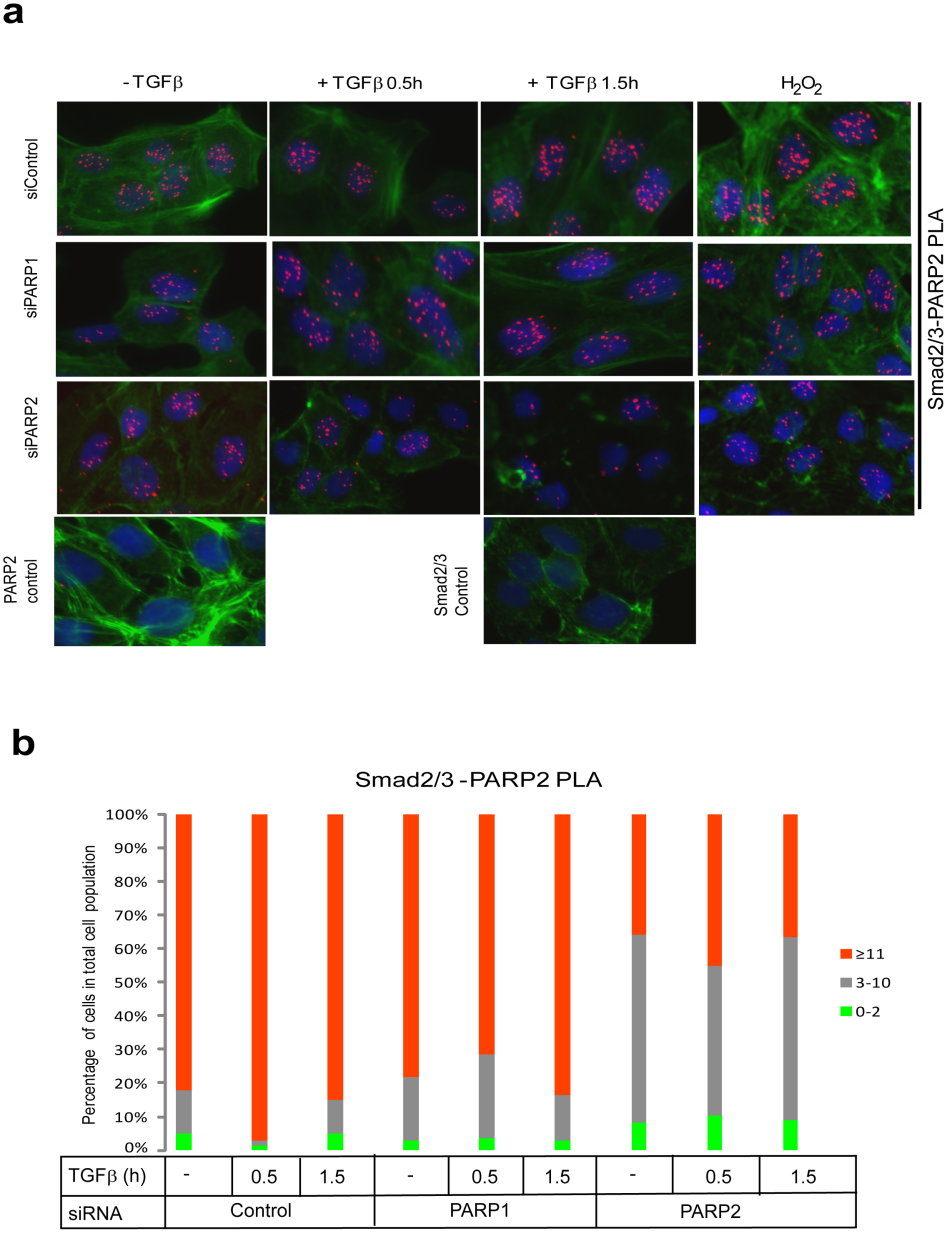
PLA of endogenous Smad2/3 and PARP-2 complexes in HaCaT cells. (**a**) HaCaT cells were analyzed with PLA using antibodies against Smad2/3 and PARP-2 after transfection with control or the indicated specific siRNAs and stimulation with vehicle (-TGFβ) or with 2 ng/ml TGFβ1 for the indicated time periods. Specific RCA signals were detected in the nuclei. Cells stimulated with 10 mM hydrogen peroxide for 10 min served as positive control. PLA with single antibodies against Smad2/3 or PARP-2 are shown as controls. PLA images are shown as in Fig. 1a. (**b**) Quantification of the experiment shown in panel (a) following the histogram method of Fig. 1b. The figure shows a representative experiment from three or more repeats.

Formation of endogenous complexes between PARP-2 and R-Smads using the PLA approach in HaCaT cells after TGFβ or peroxide treatment was also studied (Fig. 3). Once more, PLA-positive RCA products were detected in the nucleus. The incidence of R-Smad/PARP-2 complexes was higher after TGFβ stimulation especially at 0.5 h and lower after 1.5 h (Fig. 3), and persisted even up to 6 h after TGFβ stimulation (data not shown), while they were also increased by peroxide treatment (Fig. 3a). The negative controls of PLA with single antibodies and silencing of PARP-2 with the siRNA showed high degree of specificity in the analysis (Fig. 3). Interestingly, when the endogenous PARP-1 was silenced the R-Smad/PARP-2 complexes were significantly but not dramatically decreased (Fig. 3b), suggesting that PARP-1 only partly contributes to the formation of the complex between PARP-2 and R-Smad.

**Figure 4:**
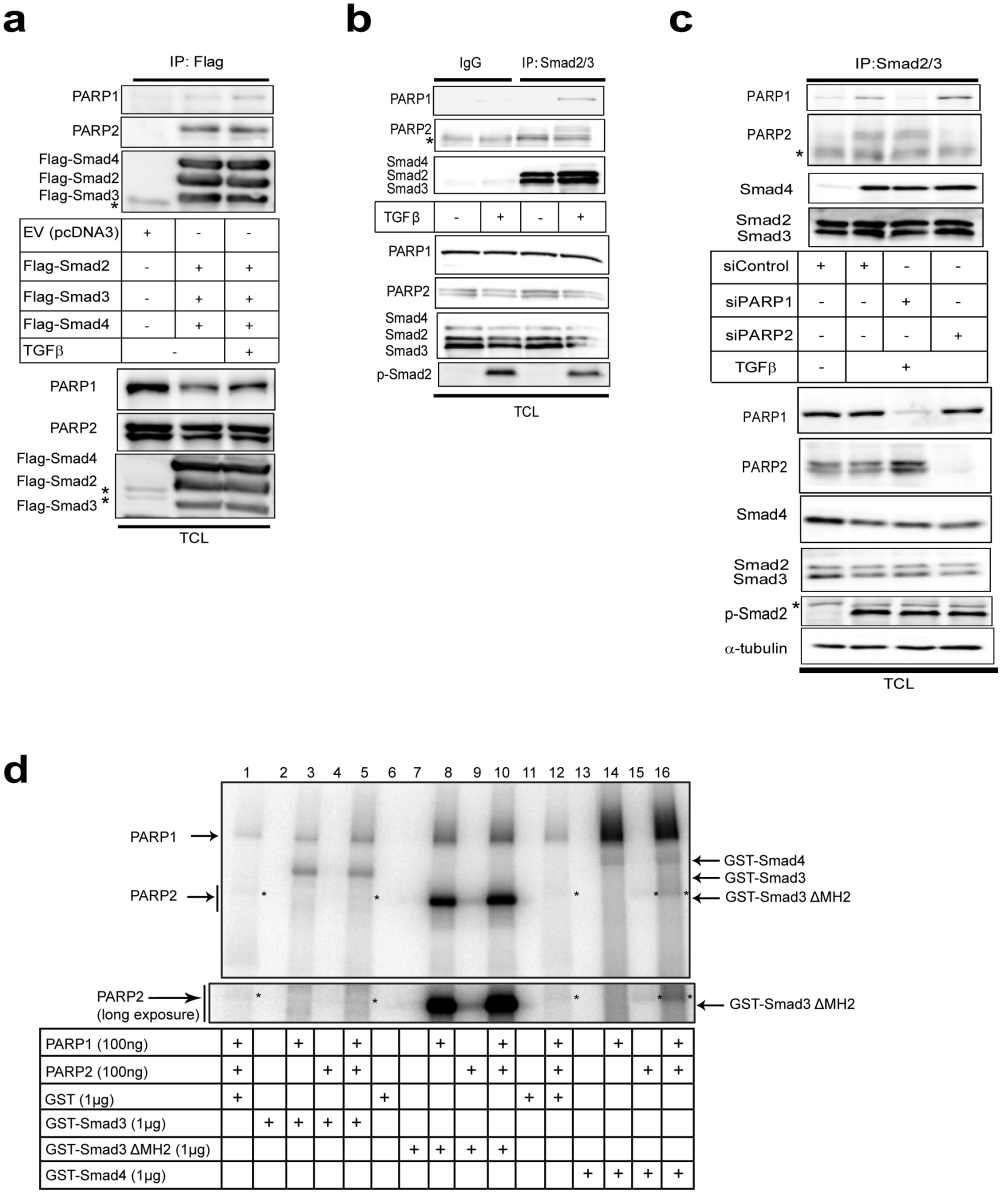
TGFβ induces formation of endogenous complexes between Smads and PARP-1/2 in HaCaT cells. (**a**) Immunoprecipitation of Flag-Smad2/3/4 followed by immunoblotting for PARP-1 and PARP-2 in cell lysates of transiently transfected HEK-293T cells with the indicated plasmids and after stimulation with vehicle (-TGFβ) or 5 ng/ml TGFβ1 for 30 min. Expression levels of all transfected proteins and endogenous PARP-1 and PARP-2 are shown in the total cell lysate (TCL) immunoblot of the HEK 293T cells. PARP-1 immunoblot also serves as protein loading control. Stars mark non-specific protein bands. (**b**) Immunoprecipitation of Smad2/3 followed by immunoblotting for PARP-1, PARP-2, Smad2/3 and Smad4 in HaCaT cells stimulated with vehicle (-TGFβ) or with 5 ng/ml TGFβ1 for 30 min. Negative control immunoprecipitation using non-specific IgG is shown. TCL shows the levels of endogenous proteins before immunoprecipitation. PARP-1 immunoblot also serves as protein loading control and C-terminal phospho-Smad2 (p-Smad2) serves as control for the efficiency of stimulation of TGFβ signaling. (**c**) Immunoprecipitation of Smad2/3 followed by immunoblotting for PARP-1, PARP-2, Smad2/3 and Smad4 in HaCaT cells transfected with the indicated siRNAs and stimulated with 5 ng/ml TGFβ1 for 30 min or not (-TGFβ). Efficiency of knockdown of PARP-1 and PARP-2, total Smad levels, phospho-Smad2 levels and protein loading (α-tubulin) controls can be seen in the TCL. (**d**) In vitro PARylation assay after glutathion-pulldown of control GST protein or GST-Smad3, truncated mutant of GST-Smad3 (ΔMH2) and GST-Smad4 in the presence of recombinant PARP-1 and/or recombinant PARP-2 as indicated. A star (weak signal) indicates the position of PARP-2 in addition to the arrow. A longer exposure of the autoradiogram around the migrating position of PARP-2 is shown at the bottom. Note the position of ADP-ribosylated Smad proteins that migrate at the size of the core non-ADP-ribosylated proteins. The input amounts of recombinant proteins were calculated based on staining of test SDS-PAGE with CBB as shown in Fig. S1. The figure shows results from representative experiments that were repeated at least twice.

Subsequently, we studied protein interactions by performing immunoprecipitation assays in embryonic kidney cells under conditions where all three Smad proteins (Smad2, Smad3 and Smad4) were overexpressed at stoichiometric levels to simulate endogenous Smad signaling (Fig. 4a). We have found that expression of all three Smads leads to the formation of robust levels of Smad complexes and probing the cells with antibodies against the phosphorylated C-terminal of Smad2 or Smad3 indicated strong activation of these Smads, as if the cells produced autocrine TGFβ (data not shown). Both endogenous PARP-1 and PARP-2 were co-precipitated with the three Smads. The PARP-2 antibody used recognized two near migrating protein bands (Fig. 4a) that both represent PARP-2 protein as both are lost after PARP-2-specific silencing (Fig. 4c). Interestingly only the slower migrating PARP-2 species co-precipitated with the Smads, while the faster migrating PARP-2 protein species showed weak association with the Smads (Fig. 4a). We currently do not understand the reason behind this observation.

We also detected endogenous complexes between R-Smad (Smad2/3) and PARP-1 and PARP-2 in HaCaT cells that were used for the PLA analysis (Fig. 4b). In this endogenous co-precipitation, PARP-1 formed complexes with R-Smads only after 0.5 h stimulation with TGFβ (Fig. 4b). PARP-2 associated with R-Smads even without TGFβ stimulation, but its association was enhanced after stimulation (Fig. 4b). Immunoblotting with a Smad4 antibody revealed the TGFβ-dependent association of endogenous Smad4 with Smad2/3, serving as positive control of functional TGFβ signaling (Fig. 4b). Use of an isotype-matched control immunoglobulin (IgG) for the immunoprecipitation demonstrated very low level of co-precipitating non-specific proteins binding to the Smads (Fig. 4b). By performing the siRNA-mediated knockdowns of each PARP protein, as done in the PLA assay (Fig. 2, 3), we confirmed that TGFβ signaling promotes distinct complexes of R-Smads with PARP-1 and with PARP-2, as well as with Smad4, the positive control for signaling (Fig. 4c). Thus, silencing 80–90% of PARP-1 caused loss of R-Smad/PARP-1 complexes, but did not affect the R-Smad/PARP-2 complexes. Similarly, loss of 90% of PARP-2 did not affect the R-Smad/PARP-1 complexes (Fig. 4c). It is worth noting that by comparing PLA (Fig. 2, 3) with co-immunoprecipitation assays (Fig. 4b, c), it appears as TGFβ is strongly required for formation of endogenous R-Smad/PARP complexes as judged by co-precipitation assay (Fig. 4b, c), while such complexes occur also in the absence of TGFβ stimulation as judged by PLA (Fig. 2, 3). This may reflect the fact that PLA measures proximity between proteins but not necessarily formation of stable complexes, whereas the co-precipitation assay, especially after stringent washes with salt, measures the formation of more stable protein complexes. Furthermore, this difference could also indicate that the phosphorylation of Smads leads to a stronger and more stable interaction with PARP1 and PARP2 that better endures the immunoprecipitation protocol. We conclude that TGFβ signaling rapidly promotes R-Smad/PARP1 and R-Smad/PARP-2 complexes that reside in the nucleus.

### Induction of ADP-ribosylation by Smad proteins

The in vivo ADP-ribosylation of endogenous Smad3 (Fig. 1) and the endogenous complexes between R-Smad and PARP-1/2 (Fig. 2–4) prompted further in vitro experiments. We previously reported that Smad3 and Smad4 are ADP-ribosylated by PARP-1 and also enhance auto-ADP-ribosylation of PARP-1 in vitro [9]. We now tested the capacity of purified Smad proteins to associate with PARP-1 and PARP-2 and become poly(ADP-ribosyl)ated, using in vitro ADP-ribosylation assays (Fig. 4d). Recombinant GST-Smads isolated from *E. coli* (Fig. S1) and insect cell-derived PARP-1 and PARP-2 purified after baculovirus infection were added in reactions together with radioactive β-NAD, which served as the tracer that can reveal ADP-ribosylation on any of the proteins included in the reaction after separation on SDS-PAGE (Fig. 4d). In addition, since the Smad proteins used were tagged with GST, we could perform glutathione-based pull down assays followed by SDS-PAGE, which allowed us to monitor ADP-ribosylated proteins simultaneously with their ability to form complexes and co-precipitate together (Fig. 4d). In these experiments we tested three specific Smad variants, full length Smad3 N-terminally fused to GST, GST-Smad3 lacking its C-terminal Mad homology 2 (MH2) domain (GST-Smad3 ΔMH2) and full length GST-Smad4. The proteins were mixed in the same reaction vessel, incubated with radioactive β-NAD for 30 min and then proteins were precipitated; after washing, the samples were resolved by SDS-PAGE followed by autoradiography.

Using PARP-1 and PARP-2 together with GST as control, we observed only weak poly(ADP-ribosyl)ation of PARP-1, and very low levels of PARP-2 poly(ADP-ribosyl)ation (Fig. 4d, lanes 1, 12; stars indicate PARP-2 migration). Co-incubation of PARP-1 with GST-Smad3 led to a robust ADP-ribosylation of Smad3 (Fig. 4d, lane 3) as previously established [9], and reproduced the enhanced complex formation and activation of PARP-1 poly(ADP-ribosyl)ation (Fig. 4d, compare the PARP-1 band in lanes 1 and 3). Addition of PARP-2 in the reaction together with PARP-1 and GST-Smad3 did not enhance Smad3 ADP-ribosylation but led to weak but detectable and reproducible poly(ADP-ribosyl)ation of PARP-2 (Fig. 4d, lane 5). Similar results were obtained with GST-Smad3 ΔMH2 (Fig. 4d, lanes 8–10), however, PARP-2 migrated exactly at the same position as GST-Smad3 ΔMH2 prohibiting us from observing effects on PARP-2 ADP-ribosylation; moreover, this deletion mutant led to detection of a more robust poly(ADP-ribosyl)ation of PARP-1 and itself, as previously described [9], due to the tighter association of the N-terminal Smad3 domain (MH1) with PARP-1. Interestingly, when GST-Smad4 was incubated with PARPs, we observed ADP-ribosylation of Smad4, but less efficient than the ADP-ribosylation of Smad3 as previously explained [9]. However, Smad4 led to more efficient detection of auto-poly(ADP-ribosyl)ation of PARP-1 than Smad3 (see thick smear migrating upwards in Fig. 4d, lanes 14, 16) and the poly(ADP-ribosyl)ation of PARP-2 was correspondingly enhanced (see long exposure in Fig. 4d). PARP-2 alone did not ADP-ribosylate Smads (Fig. 4d, lanes 4, 9, 15). As a control, excess amount of GST protein did not co-precipitate ADP-ribosylated proteins, neither did GST become ADP-ribosylated (Fig. 4d, lanes 1, 12).

The above experiments reconfirmed our previous results that Smad3 and Smad4 can be directly ADP-ribosylated by PARP-1, and of the ability of Smad3 or Smad4 to stimulate interaction and activation of PARP-1 auto-poly(ADP-ribosyl)ation. The data further demonstrate that Smads also bind and activate PARP-2, albeit much less efficiently. These in vitro experiments also suggest that purified PARP-1 is more catalytically active than purified PARP-2, as previously reported [18], and do not allow us to fully conclude whether the observed ADP-ribosylation of PARP-2 in the presence of PARP-1 and Smads is due to the activity of PARP-1 or PARP-2 itself. However, the weak but detectable auto-poly(ADP-ribosyl)ation of PARP-2 in experiments where PARP-1 was left out and Smad4 was co-incubated (Fig. 4d, lane 15) suggests that PARP-2 can exhibit genuine ADP-ribosylation activity, which is assisted by the presence of Smad4. We therefore conclude that one possible function of the observed protein complex between Smads, PARP-1 and PARP-2, is that the binding of Smads regulates or stabilizes the catalytically active form of these enzymes.

### Impact of TGFβ on formation of nuclear PARP-1/PARP-2 complexes and their ADP-ribosylation

**Figure 5:**
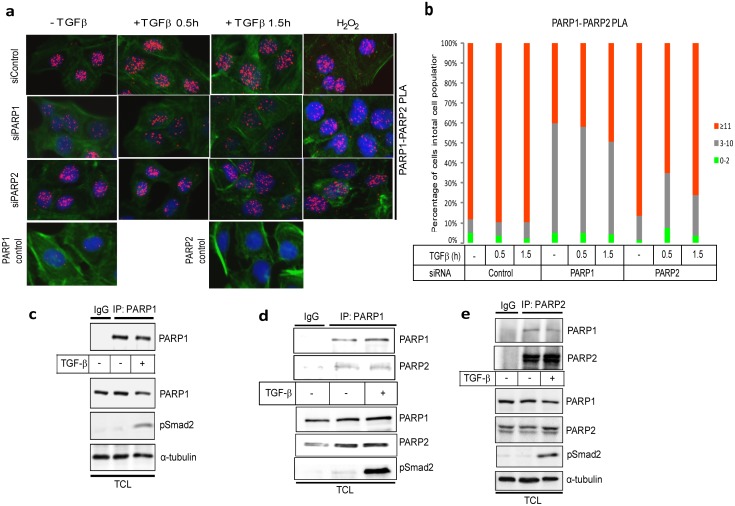
Analysis of endogenous PARP-1 and PARP-2 complexes in HaCaT cells. (**a**) HaCaT cells were analyzed with PLA using antibodies against PARP-1 and PARP-2 after transfection with control or the indicated specific siRNAs and stimulation with vehicle (-TGFβ) or with 2 ng/ml TGFβ1 for the indicated time periods. Specific RCA signals were detected in the nuclei. Cells stimulated with 10 mM hydrogen peroxide for 10 min served as positive control. PLA with single antibodies against PARP-1 or PARP-2 are shown as controls. PLA images are shown as in Fig. 1a. (**b**) Quantification of the experiment shown in panel (a) following the histogram method of Fig. 1b. Panels a–b show a representative experiment from three or more repeats. (**c**) Immunoprecipitation of PARP-1 followed by immunoblotting for PARP-1 in HaCaT cells stimulated with vehicle (-TGFβ) or with 5 ng/ml TGFβ1 for 30 min. Negative control immunoprecipitation using non-specific IgG is shown. TCL shows the levels of endogenous proteins before immunoprecipitation. C-terminal phospho-Smad2 (p-Smad2) serves as control for the efficiency of stimulation of TGFβ signaling and α-tubulin as protein loading control. (**d**) Immunoprecipitation of PARP-1 followed by immunoblotting for PARP-1 and PARP-2 in HaCaT cells stimulated with vehicle (-TGFβ) or with 5 ng/ml TGFβ1 for 30 min. Negative control immunoprecipitation using non-specific IgG is shown. TCL shows the levels of endogenous proteins before immunoprecipitation. PARP-1 immunoblot also serves as protein loading control and C-terminal phospho-Smad2 (p-Smad2) serves as control for the efficiency of stimulation of TGFβ signaling. (**e**) Reciprocal immunoprecipitation of PARP-2 followed by immunoblotting for PARP-1 and PARP-2 in HaCaT cells stimulated with vehicle (-TGFβ) or with 5 ng/ml TGFβ1 for 30 min. Negative control immunoprecipitation using non-specific IgG is shown. TCL shows the levels of endogenous proteins before immunoprecipitation. C-terminal phospho-Smad2 (p-Smad2) serves as control for the efficiency of stimulation of TGFβ signaling and α-tubulin as protein loading control. Panels c–e show results from representative experiments that were repeated at least twice.

Based on the previously established association of PARP-1 with PARP-2 [18], and our evidence that TGFβ can induce nuclear poly(ADP-ribosyl)ation activity ([9] and Fig. 1), we tested whether TGFβ also affects the complex between the two nuclear PARPs. PLA using PARP-1 and PARP-2 antibodies in HaCaT keratinocytes showed exclusively nuclear PARP-1/PARP-2 protein complexes, as expected (Fig. 5a, b). Stimulation of the cells with TGFβ for 0.5 or 1.5 h led to a weak but reproducible increase of nuclear RCA signals especially at 1.5 h (Fig. 5a, b). As a control, peroxide treatment enhanced the nuclear PARP-1/PARP-2 complexes even further (Fig. 5a). Silencing of PARP-1 reduced the number of complexes significantly (Fig. 5a, b). Silencing PARP-2 also reduced the number of nuclear complexes, albeit not so efficiently (Fig. 5a, b). The loss of PLA-positive signals in these experiments reflected rather well the silencing efficiency, which was approximately 80% for PARP-1 and only 60% for PARP-2 (not shown). Controls with single PARP-1 or PARP-2 antibodies gave the anticipated low background signals (Fig. 5a).

The PLA experiments were reproduced using co-immunoprecipitation assays in the same cell system, measuring the endogenous complexes of PARP-1 and PARP-2 in HaCaT cells (Fig. 5c–e). First, we established the efficient immunoprecipitation by the PARP-1 antibody (Fig. 5c). Stimulation with TGFβ did not affect at all the efficiency of immunoprecipitation of PARP-1 as revealed by immunoblot with the same antibody (Fig. 5c). Then, by immunoprecipitating first PARP-1 or PARP-2 followed by immunoblotting with the reciprocal antibody gave evidence for the presence of PARP-1/PARP-2 complexes that were only weakly affected by TGFβ stimulation (Fig. 5c, d), as predicted from the PLA results (Fig. 5a, b). Use of an isotype-matched control immunoglobulin (IgG) for the immunoprecipitation gave only low amounts of co-precipitating proteins (Fig. 5a, b).

**Figure 6:**
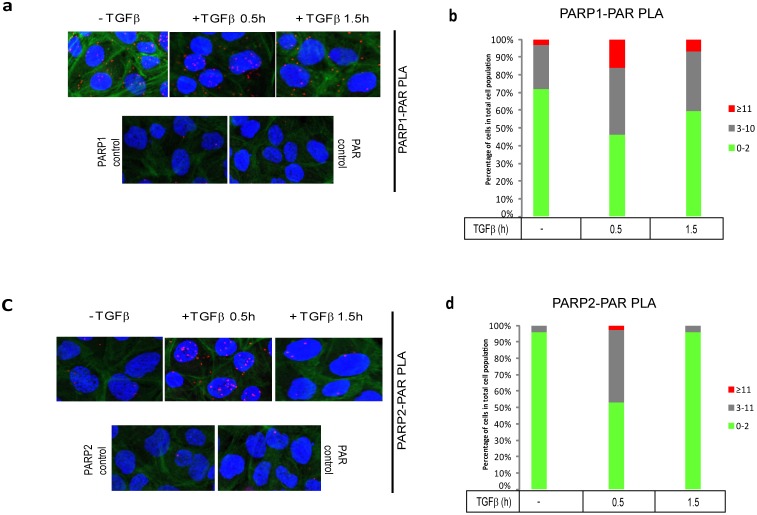
PLA of endogenous PARP-1 and PARP-2 ADP-ribosylation after TGFβ stimulation in HaCaT cells. (**a, c**) HaCaT cells were analyzed with PLA using antibodies against PARP-1 and PAR chains (**a**) or antibodies against PARP-2 and PAR (**c**) after stimulation with vehicle (0 min) or with 2 ng/ml TGFβ1 for the indicated time periods. Specific RCA signals were detected in the nuclei. PLA with single antibodies against PARP-1 or PAR are shown as controls. PLA images are shown as in Fig. 1a. (**b, d**) Quantification of the experiments shown in panels (a, c) following the histogram method of Fig. 1b. The figure shows a representative experiment from three or more repeats.

We then performed in situ PLA for PARP-1 and PARP-2 ADP-ribosylation and measured effects of TGFβ stimulation (Fig. 6). In contrast to endogenous Smad3, which showed weak basal levels of ADP-ribosylation using the PLA (Figure 1), endogenous PARP-1 in the same cells, showed rather high level of RCA signals, compatible with an active PARP-1 enzyme that was ADP-ribosylated (Fig. 6a, b). Under the same conditions, PARP-2 showed weaker than PARP-1 but higher than Smad3 ADP-ribosylation (Fig. 6c, d). Stimulation with TGFβ for 30 min resulted in measurable enhancement of ADP-ribosylation of PARP-1 and even more dramatic enhancement of ribosylation of PARP-2 (Fig. 6b,d). At 90 min after TGFβ stimulation ADP-ribosylation of both proteins decreased and especially for PARP-2 reached the same low levels as in control, unstimulated cells (Fig. 6b,d). We therefore conclude that PARP-1 and PARP-2 complexes exist in the nucleus, and TGFβ either does not influence or only weakly affects this association, whereas TGFβ prominently promotes complexes of each PARP protein with Smads, and also promotes ADP-ribosylation of both PARP enzymes.

### Impact of PARP-2 on TGFβ-regulated gene expression

**Figure 7:**
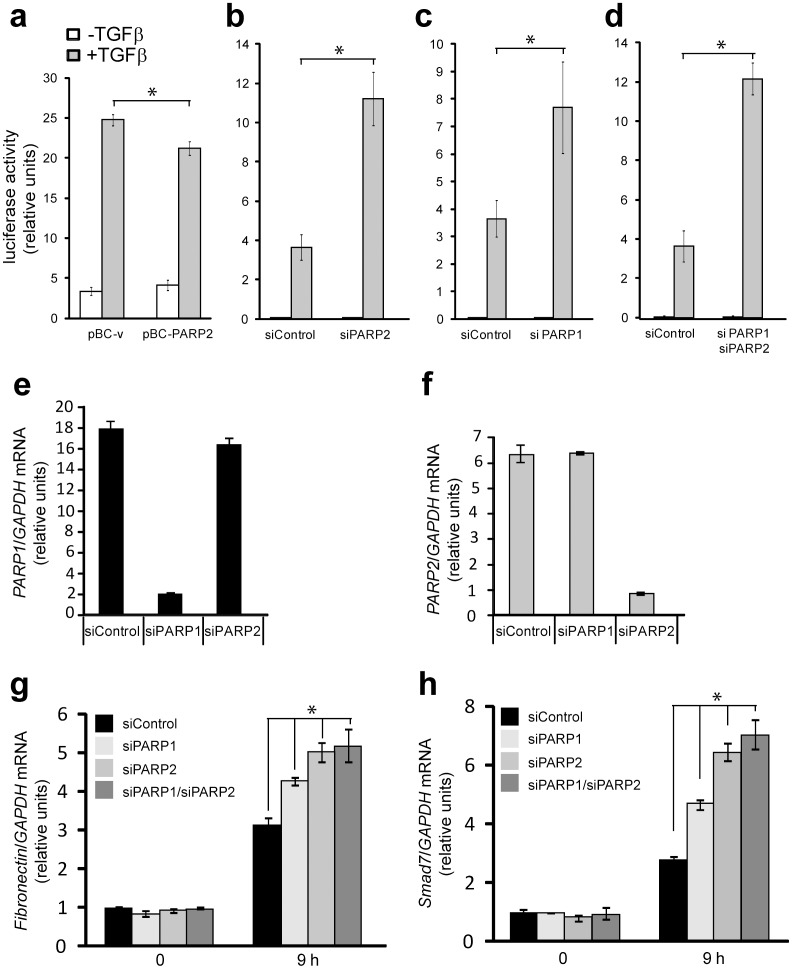
Regulation of gene expression by PARP-1 and PARP-2 during TGFβ signaling. (**a**) CAGA_12_ promoter luciferase assay in HaCaT cells transiently transfected with pBC-vector (pBC-v) and pBC-PARP-2 and stimulated (grey bars) or not (white bars) with 5 ng/ml TGFβ1 for 24 h. Average values with standard errors of luciferase activity normalized to the corresponding co-transfected β-galactosidase activity from triplicate determinations are shown based on a representative single experiment. (**b–d**) CAGA_12_ promoter luciferase assays were performed as in panel (a) in HaCaT cells transiently transfected with the indicated siRNAs 48 h prior to stimulations with TGFβ1. Stars (panels a–d) indicate statistical significance relative to the Control condition stimulated with TGFβ1, *p*<0.05. (**e, f**) Quantitative real-time RT-PCR assays for PARP-1 (e, black bars) and PARP-2 (f, grey bars) in HaCaT cells after transient transfection with control and specific siRNAs. The specific mRNA amounts were normalized to the expression level of the housekeeping gene *GAPDH* and are expressed as relative fold-differences. Average values from triplicate determinations are shown with standard deviations as error bars. (**g, h**) Real-time RT-PCR analysis of endogenous *fibronectin* (panel g) and *Smad7* (panel h) mRNAs normalized to the corresponding *GAPDH* mRNA from human HaCaT cells transfected with the indicated siRNAs and left unstimulated (0 h) or were stimulated with 2 ng/ml TGFβ1 for 9 h. Average values from triplicate determinations and the corresponding standard errors are graphed. Stars (panels g, h) show statistical significance relative to the siControl condition stimulated with TGFβ1, *p*<0.05. The figure shows representative experiments from four or more repeats.

Since PARP-2 and PARP-1 reside in the nucleus and we previously established that PARP-1 affects the transcriptional activity of Smads [9], we hypothesized that PARP-2 should be implicated in the same process. To investigate this possibility, we performed Smad-specific promoter-luciferase assays in cells where PARP-2 was either overexpressed or silenced by siRNA (Fig. 7a, b). PARP-2 overexpression led to a weak but reproducible reduction of the Smad3/Smad4-specific CAGA_12_-luciferase promoter (Fig. 7a). Conversely, silencing of endogenous PARP-2 almost tripled the response of the same promoter to TGFβ (Fig. 7b). The impact of PARP-2 silencing on the promoter activity was as pronounced as that of PARP-1 silencing (Fig. 7c). Finally, silencing of both PARP-1 and PARP-2 had a similar positive effect on promoter activity (Fig. 7d), however, we never observed additive or synergistic effects when the two PARPs were silenced.

The CAGA_12_-luciferase reporter provides an easy tool to assay directly the transcriptional activity of Smads. Endogenous regulatory sequences of various genes that respond to TGFβ are more complex and depend on the activity of Smad complexes, interacting transcription factors and many cooperating chromatin modulators and co-activators/co-repressors [3]. For this reason, the impact of PARP silencing on gene expression in response to TGFβ is more variable, gene-specific and cell context-specific [9], [11]. This is corroborated by our efforts in measuring the impact of PARP-2 on TGFβ target genes after siRNA-mediated silencing of PARP-2 (Fig. 7g, h). We first established siRNA transfection conditions that showed specific silencing of PARP-2 without affecting PARP-1 expression and silencing of PARP-1 without any impact on PARP-2 expression, as assessed by quantitative RT-PCR analysis (Fig. 7e, f). Under these conditions we measured the responsiveness of classic gene targets of TGFβ/Smad signaling, like *fibronectin* and *Smad7* (Fig. 7g, h). PARP-1 silencing enhanced the response of both genes when measured after 9 h of TGFβ stimulation, while PARP-2 silencing led to more robust enhancement of the gene response. Silencing of both PARP-1 and PARP-2 had almost the same effect on gene expression in response to TGFβ as PARP-2 silencing alone (Fig. 7g, h). We therefore conclude that PARP-2, like PARP-1, can play a negative regulatory role in TGFβ signaling.

### PARG interacts with Smads and de-ADP-ribosylates Smad3

**Figure 8:**
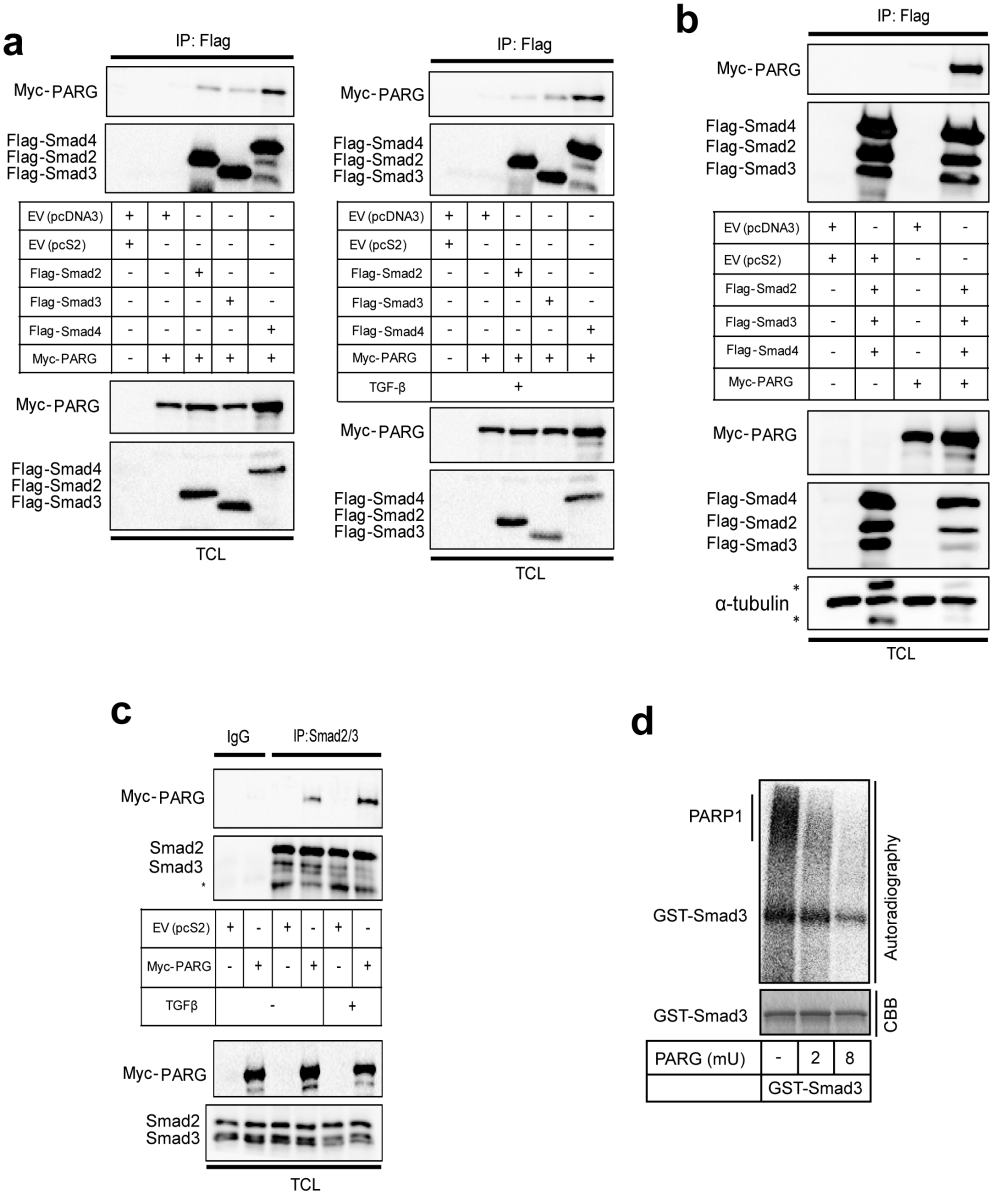
PARG forms complexes with Smad proteins and de-ADP-ribosylates Smad3. (**a**) Immunoprecipitation of Flag-Smad2, Flag-Smad3 or Flag-Smad4 followed by immunoblotting for myc-PARG in cell lysates of transiently transfected 293T cells with the indicated plasmids and after stimulation with vehicle (-TGFβ, left panel) or 5 ng/ml TGFβ1 for 30 min (right panel). Expression levels of all transfected proteins are shown in the TCL immunoblot of the 293T cells. (**b**) Immunoprecipitation of Flag-Smad2/3/4 followed by immunoblotting for myc-PARG in cell lysates of transiently transfected 293T cells with the indicated plasmids and in the absence of stimulation with TGFβ. Expression levels of all transfected proteins are shown in the TCL immunoblot of the 293T cells. α-Tubulin immunoblot serves as protein loading control. Stars mark non-specific protein bands. (**c**) Immunoprecipitation of endogenous Smad2/3 followed by immunoblotting for transfected myc-PARG in 293T cells stimulated with vehicle (-TGFβ) or with 5 ng/ml TGFβ1 for 30 min. Negative control immunoprecipitation using non-specific IgG is shown. TCL shows the levels of endogenous Smad2/3 proteins and transfected myc-PARG before immunoprecipitation. Smad2/3 immunoblot also serves as protein loading control. (**d**) In vitro de-ADP-ribosylation assay of Smad3 using PARG. GST-Smad3 was first ADP-ribosylated using recombinant PARP-1. The proteins were pulled-down and washed, prior to reconstitution with PARG reaction buffer and increasing amounts of recombinant PARG (shown as milli-units (mU) of enzymatic activity). The ADP-ribosylated proteins are shown in the autoradiogram along with the CBB-stained input GST-Smad3 levels. Panels a–c show results from representative experiments that were repeated at least twice and panel d shows results from representative experiments that were repeated at least three times.

We then shifted our attention to the possibility that Smad ADP-ribosylation is reversible. First, we asked whether PARG can form complexes with the three Smads of the TGFβ pathway (Fig. 8). We could not identify a reliable antibody that could detect endogenous PARG levels in our cells, and thus, we transfected myc-tagged PARG in 293T cells together with each of the Flag-tagged Smad2, Smad3 and Smad4 (Fig. 8a). Each one of the three Smads showed specific co-immunoprecipitation with myc-PARG (Fig. 8a, left panel). Stimulation of cells with TGFβ resulted in a weak but reproducible enhancement of the complex between Smad3 and PARG and between Smad4 and PARG (Fig. 8a, right panel). Co-expression of all three Smads also showed the same robust co-precipitation of PARG in the same cell system (Fig. 8b). Immunoprecipitation of endogenous Smad2/3 from 293T cells resulted in efficient co-precipitation of the transfected myc-PARG, which was further enhanced after stimulation with TGFβ (Fig. 8c). These experiments demonstrate that PARG has the potential to form complexes with Smad proteins of the TGFβ pathway.

We then investigated how the Smad ADP-ribosylation pattern is affected by increasing β-NAD levels. We incubated GST-Smad3 together with PARP-1 and radiolabeled β-NAD; pull-down of the bound proteins followed by electrophoresis and autoradiography resulted in detectable ADP-ribosylated Smad3 (radioactive band of the same size as GST-Smad3), as well as bound auto-poly(ADP-ribosyl)ated PARP-1 appearing as a high molecular weight smear migrating slower than the core PARP-1 protein (Fig. S2, lane 1). We then used a constant amount of radioactive β-NAD and increasing concentrations of unlabeled β-NAD (Fig. S2, lanes 1–4). We observed ADP-ribosylation of GST-Smad3 under all β-NAD concentrations. Increasing the concentration of unlabeled β-NAD enhanced ADP-ribosylation of GST-Smad3 and PARP-1 (Fig. S2, lane 2), but at higher concentrations the high amount of unlabeled β-NAD diluted the radiolabeled tracer and we recorded a loss in signal (Fig. S2, lanes 3, 4). As expected, PARP-1 shifted upwards in size with increasing amounts of β-NAD (Fig. S2, top smear, lanes 2–3), illustrating the ability of PARP-1 to become poly(ADP-ribosyl)ated at one or several sites. At the highest concentrations of non-radiolabeled β-NAD, ^32^P-ADP-ribosylation signals were competed out from PARP-1 to a large extent, due to the dilution effect mentioned above. In contrast to the smear of auto-poly(ADP-ribosyl)ated PARP-1 there was no shift in size of ADP-ribosylated GST-Smad3 despite the increased concentrations of β-NAD, only competition and loss of the sharp radiolabeled GST-Smad3 protein band could be observed (Fig. S2). This suggests that, under in vitro conditions, PARP-1 mainly oligo(ADP-ribosyl)ates GST-Smad3 at one or a limited number of sites since excess of β-NAD fails to reveal high molecular size smears.

Next, we tested whether PARG could de-ADP-ribosylate Smad3 by first performing ADP-ribosylation reactions with PARP-1 and GST-Smad3 as substrates, and then incubating with recombinant PARG (Fig. 8d). The reaction with PARG efficiently removed ADP-ribosylation from GST-Smad3 in a dose-dependent manner. However, the radioactive signal could not be completely removed from the core GST-Smad3 protein species, which probably reflects the inability of PARG to cleave the last ADP-ribose unit, which is coupled to the protein substrate [21]. In contrast, the larger sized smears, most likely corresponding to poly(ADP-ribosyl)ated PARP-1, were efficiently removed by PARG. In summary, the glycohydrolase PARG can effectively process the added poly-/oligo(ADP-ribose) units from both GST-Smad3 and PARP-1, but fails to act as a mono(ADP-ribose) hydrolase as predicted from previous studies [22].

### Endogenous PARP-1 and PARG have opposing roles on TGFβ-induced gene expression

**Figure 9:**
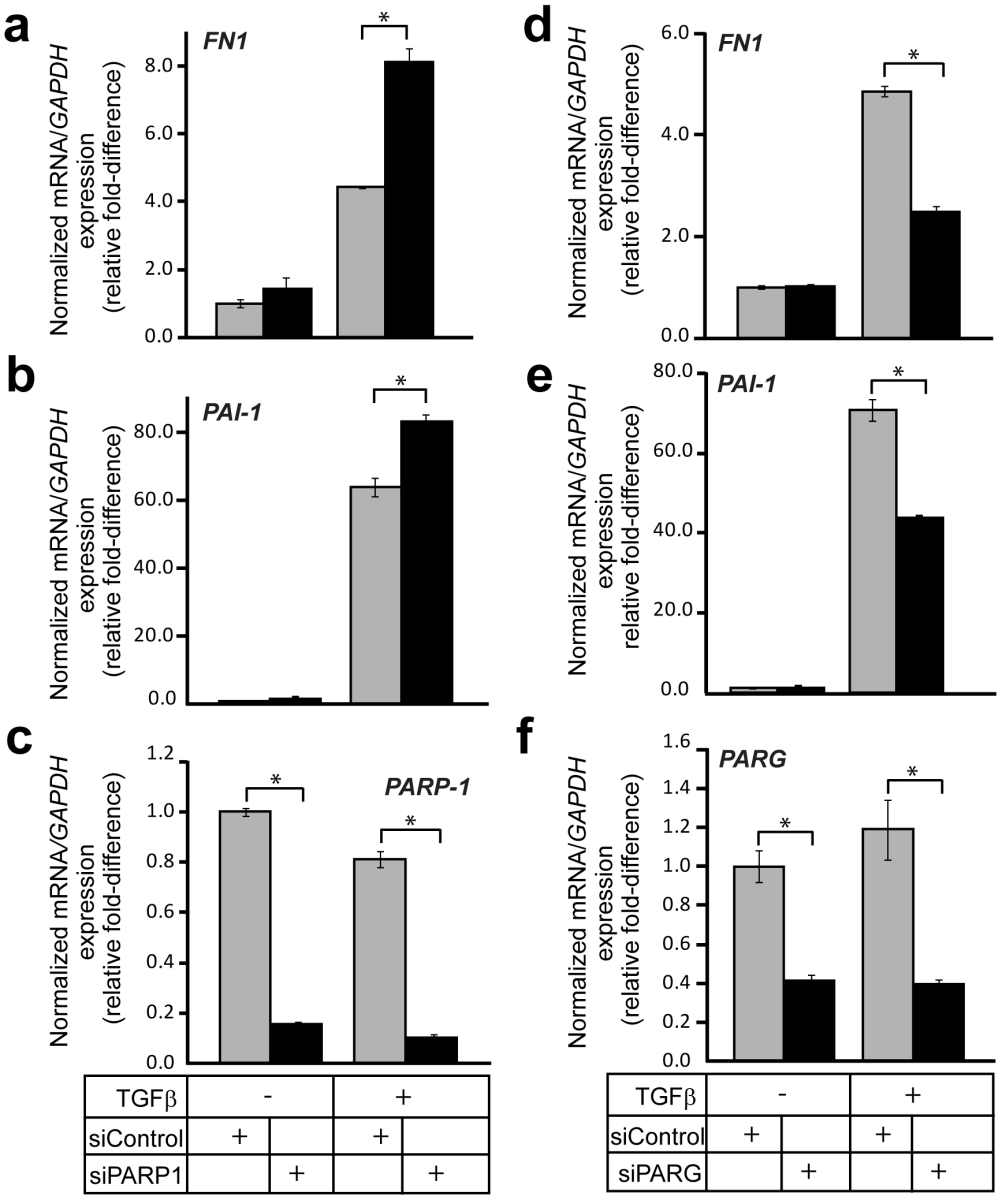
PARG regulates transcriptional responses to TGFβ. (**a–c**) Real-time RT-PCR analysis of endogenous *fibronectin (FN1)* (a), *PAI-1* (b) and control *PARP-1* (c) mRNAs in HaCaT cells transiently transfected with the indicated siRNAs (bottom of panel c) prior to stimulation (or not) with 5 ng/ml TGFβ1 for 9 h. The data are graphed as in Fig. 7g, h. (**d–f**) Real-time RT-PCR analysis of endogenous *fibronectin (FN1)* (d), *PAI-1* (e) and control *PARG* (f) mRNAs in HaCaT cells transiently transfected with the indicated siRNAs (bottom of panel f) prior to stimulation (or not) with 5 ng/ml TGFβ1 for 9 h. The data are graphed as in Fig. 7g, h. Stars (panels b–g) indicate statistical significance, *p*<0.05. The figure shows representative experiments from four or more repeats.

The evidence that PARG can de-ADP-ribosylate Smad3 *in vitro* made us design experiments to test for possible effects that endogenous PARG has on signaling. We compared TGFβ-induced gene expression after performing knock-down of either endogenous PARP-1 or PARG. As shown previously [9], depleting PARP-1 led to a significant elevation of TGFβ-induced expression of endogenous *fibronectin* (*FN1*) and *PAI-1* mRNA after 9 h of stimulation (Fig. 9a, b). Knockdown of endogenous PARP-1 was verified at the mRNA level (Fig. 9c). Interestingly, depleting PARG had the opposite effect on mRNA accumulation of these two genes; the induction of either *fibronectin* or *PAI-1* expression by 9 h stimulation with TGFβ was significantly reduced when PARG expression was silenced (Fig. 9d, e). Knockdown efficiency of endogenous PARG was determined by RT-PCR (Fig. 9f).

We also checked whether the hampered TGFβ-mediated gene induction seen after silencing PARG expression also had an impact on the corresponding induced protein levels. Indeed, when PARG expression was silenced, the fibronectin and PAI-1 protein levels were induced to lower levels than those seen in control cells after 9 and 24 h of TGFβ stimulation (Fig. S3). The difference at 9 h of stimulation was most noticeable, while after 24 h the differences were reproducible but smaller. No major effects on TGFβ-induced phosphorylation of Smad2 were found that could account for the changes seen on downstream fibronectin and PAI-1 expression (Fig. S3). This suggests that the observed effects of endogenous PARG silencing more likely reflect regulation at the transcriptional level.

### Silencing of PARP-1 rescues the PARG-mediated reduction of TGFβ signaling

**Figure 10:**
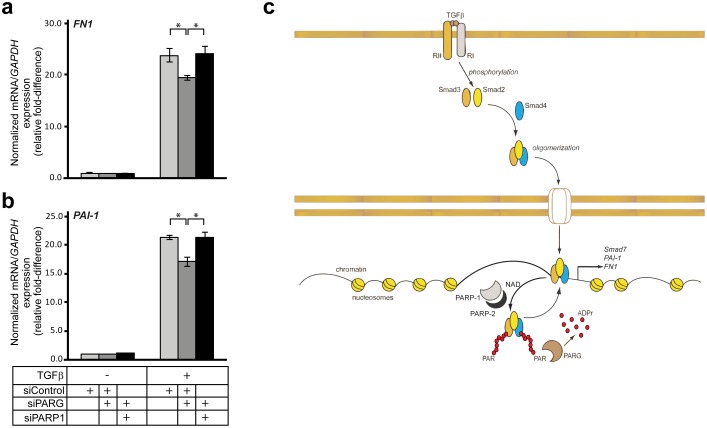
Regulation of Smad signaling by PARG and PARP-1. (**a, b**) Real-time RT-PCR analysis of endogenous *fibronectin (FN1)* (a) and *PAI-1* (b) mRNAs in HaCaT cells transiently transfected with the indicated siRNAs (bottom of panel b) prior to stimulation (or not) with 5 ng/ml TGFβ1 for 24 h. The data are graphed as in Fig. 7g, h. Stars (panels a, b) indicate statistical significance, *p*<0.05. The figure shows representative experiments from four or more repeats. (**c**) A model depicting TGFβ dimeric ligand that activates its cell surface type II (RII) and type I (RI) receptors, which phosphorylates Smad2 and Smad3, leading to oligomerization of Smad2, Smad3 and Smad4 into trimeric complexes. Smad oligomers enter the nucleus via nuclear pores and associate with chromatin in order to regulate transcription of target genes such as *Smad7*, *fibronectin (FN1)* and *PAI-1*. Nuclear PARP-1 and PARP-2 in complex associate with the Smad oligomer. For simplicity distinct complexes between Smads and PARP-1 and Smads and PARP-2 are not shown but their presence is supported by the experimental evidence. PARP-1/PARP-2 use NAD and oligo(ADP-ribosyl)ate Smad3 and Smad4 (ADP-ribose chains in red) and assist dissociation of Smads from DNA (as demonstrated in ref. [9], [10], [11]). PARG associates with ADP-ribose chains on the Smad complex and removes ADP-ribose units (ADPr) possibly generating mono(ADP-ribosyl)ated Smads (not shown). PARG therefore promotes Smad association with DNA and is required for optimal gene expression in response to TGFβ.

Since there are several factors that possess ADP-ribosylating capacity in the cell [15], and since PARG might also act through an ADP-ribosylation-independent mechanism, it was important to test if the gene expression effects, recorded by loss of PARG, were dependent on PARP-1. We designed rescue experiments where we tested if the perturbed induction of *fibronectin* and *PAI-1* mRNA by TGFβ under PARG silencing conditions could be relieved by simultaneous silencing of PARP-1. We knocked-down PARG alone or in combination with PARP-1 using the corresponding siRNAs and stimulated cells with TGFβ for 24 h (Fig. 10a, b). Depleting PARG mRNA had again a reducing effect on TGFβ-induced expression of both *fibronectin* and *PAI-1* mRNA, although the effects were significantly less after this longer (24 h) stimulation. The combination of PARG and PARP-1 siRNA could fully rescue the signal back to control levels (Fig. 10a, b). However, it did not elevate signaling beyond control levels (Fig. 10a, b), as seen when PARP-1 knockdown was performed alone (Fig. 10b, c). This suggests that PARP-1 accounts for a large part of the changes seen on TGFβ signaling after PARG knockdown; however, it is possible that other ribosylating enzymes are involved. In summary, these data establish a role of PARG as a positive mediator, or a permissive factor, that controls the transcriptional responses to TGFβ signaling.

## Discussion

The recent demonstration that TGFβ induces nuclear ADP-ribosylation, that Smad proteins associate directly with PARP-1, and that PARP-1 has regulatory impact on the functional output of the TGFβ pathway [9], [10], [11], prompted us to investigate deeper TGFβ-induced ADP-ribosylation and the role of two other known regulators of ADP-ribosylation, PARP-2 and PARG.

By using antibodies against Smad3 and poly(ADP-ribose) chains, we managed to set up a specific assay that allowed us to study Smad3 ADP-ribosylation in a timely fashion. We have previously been unable to successfully perform such analysis using immunoprecipitation assays. Interestingly, we found that TGFβ induced ADP-ribosylation of Smad3 rapidly. Specific PLA-positive RCAs were detectable already after 5 min, peaked at 10 min and were reduced to lower levels after 40–90 min (Fig. 1). This suggests that ADP-ribosylation of Smad3 is likely regulating an early nuclear event after TGFβ stimulation. We have previously reported that PARP-1 regulates Smad3/4 binding to promoter DNA by ADP-ribosylating the MH1 domain [9], [10], [11]. This observation would fit well with the timing of ADP-ribosylation, since Smads enter the nucleus and interact with promoters early on after TGFβ stimulation. Higher resolution microscopy could reveal the nuclear locations of ADP-ribosylated Smad3 and chromatin organization analysis may provide new ideas about possible functions of this molecular modification that takes place during the first minutes of TGFβ signaling. PARP-1 may prevent binding of Smads that are not yet attached to the DNA or may facilitate detachment of Smads that are already bound to promoters, whereas TGFβ-mediated activation of PARP-1 may lead to ADP-ribosylation of other proteins associated with transcription, such as transcription factors, polymerases, or histones. These interesting open questions need to be further investigated.

The analysis of PARP2 as regulator of TGFβ/Smad signaling was motivated by the current understanding that PARP-2 makes complexes with and functions in close association with PARP-1 [17]. Our results have shown that Smads form complexes with PARP-2 in the cell nucleus (Fig. 2–4), and that Smads may enhance the ADP-ribosylation of PARP-2 (Fig. 4d). We have not been able to identify unique functions of Smad/PARP-2 versus Smad/PARP-1 complexes. On the other hand, these complexes form and are not necessarily dependent on each other, i.e. Smad/PARP-1 complexes to a large approximation do not depend on PARP-2 and Smad/PARP-2 complexes do not depend on PARP-1 (Fig. 2–4). However, the complexes are not entirely independent from each other as seen in PLA experiments (Fig. 2, 3), suggesting that the complexes may become more stable when PARP-1, PARP-2 and Smads come together. Cooperation of the Smad/PARP-1/2 complexes at the level of enzymatic activity is also supported by these experiments. In addition, PARP-2 seems to negatively regulate the direct, Smad-dependent transcriptional output of TGFβ signaling, similar to PARP-1 (Fig. 7). We therefore propose that PARP-2 functions together with PARP-1 to negatively regulate nuclear and transcription-related functions of the Smad complex (Fig. 10c).

The ability of PARP-2 to interact physically with PARP-1 has been previously established [18], and the functional interplay between these two PARP family members has been well established in vitro in cell models and in vivo in mice, and under different physiological conditions [17]. Here, we have confirmed this physical association using the PLA technique [26], which provides us with the capacity to visualize the location of the PARP-1/PARP-2 complexes and also allows us to measure rather accurately the abundance of such complexes (Fig. 5). As expected, the PARP-1/PARP-2 complexes could be localized only in cell nuclei (Fig. 5a), and PLA allowed us to establish that these complexes are only weakly enhanced or stabilized upon relatively short (0.5–1.5 h) stimulation with TGFβ (Fig. 5b). This change is, however, compatible with the time frame of association of Smad proteins of the TGFβ pathway with PARP-1 and PARP-2 (Fig. 2, 3). Thus, the data suggest that when Smad complexes enter the nucleus in response to TGFβ signaling, they meet and associate with PARP-1 and PARP-2 that are already in complex with each other.

Another interesting corollary of the association between Smads and PARPs is the possible regulation of the enzymatic activity and resulting ADP-ribosylation catalyzed by the PARPs (Fig. 1, 4d). Previous reports demonstrated that TGFβ enhances ADP-ribosylation of nuclear proteins and of PARP-1 itself in cells [9], [10], [11]. The time frame of Smad3 ADP-ribosylation falls well inside the time window when Smads associate with PARP-1 and PARP-2 in the nucleus (Fig. 2, 3). Furthermore, the in vitro experiments have revealed that both Smad3 and Smad4 are capable of co-precipitating with activated poly(ADP-ribosyl)ated PARP-2 and PARP-1 (Fig. 4d). In addition, the experiments suggest that PARP-1 is required for the more effective ADP-ribosylation of PARP-2 itself. However, we cannot preclude that this is an effect due to the quality of our purified PARP-2 protein. PLA experiments aiming at measuring PARP-1 and PARP-2 ADP-ribosylation corroborate the above conclusion as TGFβ appeared to enhance ADP-ribosylation of both enzymes, and this was much more dramatic in the case of PARP-2 (Fig. 6). Interestingly, the effect of TGFβ on PARP-1 or PARP-2 ADP-ribosylation, as measured by PLA, coincided with the formation of Smad3-PARP-1/2 complexes (Fig. 2, 3). This suggests the possibility that as nuclear Smad complexes associate with PARP-1 and PARP-2, they may also enhance the ADP-ribosylation of these two proteins. Whether enhancement of PARP-1 and PARP-2 ADP-ribosylation by TGFβ was mediated by Smad3, or by the association of Smad3 with the PARP enzymes, could not yet been confirmed at the cellular level due to the relative failure of anti-PAR PLA after transfection with siRNAs or plasmids expressing cDNAs. Thus, the mechanism whereby Smads regulate ADP-ribosylation of PARPs requires deeper investigation. One possibility is that upon binding, Smads activate the catalytic activity of PARP-1 and PARP-2; alternatively, Smad binding to PARPs, exposes more effectively the auto-modification domain of PARPs, thus allowing more robust and stable ADP-ribosylation of the protein substrate. To explain these mechanistic details, deeper biochemical and structural studies are needed.

We have also focused our attention on the pattern of Smad3 ADP-ribosylation in vitro and on the action of the enzyme PARG that cleaves off PAR chains from modified proteins [21]. PARG exhibited robust complex formation with Smads of the TGFβ pathway (Fig. 8). The lack of a reliable antibody did not allow us to measure fully endogenous complexes between PARG and Smads. However, despite transfection of cells with exogenous PARG, we could observe that TGFβ stimulation promoted the association of endogenous Smad2/3 with PARG (Fig. 8c). Using recombinant PARG enzyme we then demonstrated that PARG is capable of de-ADP-ribosylating Smad3 (Fig. 8d). Furthermore, increasing the β-NAD levels in the in vitro ribosylation assays showed that Smad3 is primarily oligo(ADP-ribosyl)ated by PARP-1 (Fig. S2). This is in contrast to PARP-1 itself that is clearly poly(ADP-ribosyl)ated. Development of new technology that can more effectively measure the degree of polymerization of ADP-ribose during protein ADP-ribosylation and de-ADP-ribosylation will be essential to resolve questions regarding poly(ADP-ribose) chain length and function in an unambiguous manner.

Our observations support a model in which PARP-1, PARP-2 and PARG regulate ADP-ribosylation of Smad3 and the flow of Smad signaling (Fig. 1, 7, 8). While depletion of PARP-1 or PARP-2 led to enhancement of the transcriptional readout of TGFβ signaling (Fig. 7), depletion of PARG showed the opposite effect and significantly suppressed the amplitude of the TGFβ transcriptional response (Fig. 9). This evidence suggests that optimal and average transcriptional responses to TGFβ/Smad signaling are balanced by the action of the two opposing enzymatic activities, the ADP-ribosyl-transferases (PARP-1/2) and the ADP-ribosyl glycohydrolase PARG. Since we could not achieve complete removal of the ADP-ribose chains from Smad3 after prolonged incubation with PARG (Fig. 8d), we propose that additional enzymes may act in concert with PARG to completely de-ADP-ribosylate Smad3. Such proteins may be members of the ARH and macrodomain-containing protein families [22]. PARG has been shown to co-localize with PARP-1 along genomic sites in mammalian cells [24]. This suggests that upon entry of the Smad complex to the nucleus and formation of higher order complexes with PARP-1 and PARP-2, PARG may also be available for incorporation into such complexes in order to regulate quantitatively the degree of Smad ADP-ribosylation (Fig. 10c). Thus, nuclear PARG may constantly monitor the extent of Smad ADP-ribosylation by PARP-1/2 and provide dynamic control of the Smad-chromatin association/dissociation process (Fig. 10c). Alternatively, PARG may play a more important role at the onset of transcription in response to Smad signaling, thus guaranteeing the establishment of chromatin-bound Smad complexes. If this scenario stands true, the action of PARG may precede the action of PARP-1 during the time-dependent trajectory of Smad complexes along the chromatin.

In addition, it is worth discussing the fact that evidence from different cell systems demonstrated that PARP-1 can act either as a negative regulator of physiological responses to TGFβ, as is the case in epithelial cells (keratinocytes and mammary cells) [9] and CD4-positive T cells [10], or as a positive regulator of TGFβ responses, as is the case in vascular smooth muscle cells [11]. Our new data on the functional role of PARP-2 and PARG during regulation of TGFβ-mediated gene expression in keratinocytes supports the negative role of PARP-1 and PARP-2 and the positive role of PARG on such cellular responses (Fig. 7, 9, 10). It will be of importance to explain the molecular mechanism behind this apparent cell context-dependency. All studies so far agree that PARP-1 ADP-ribosylates Smad3 [9], [10], [11], and our new evidence suggests that Smad3 can also be de-ADP-ribosylated (Fig. 8). We therefore propose that depending on the cell type, the chromatin configuration on various genes that are destined to respond to TGFβ/Smad signaling interpret the molecular signal of Smad3 ADP-ribosylation and de-ADP-ribosylation in distinct ways. This is compatible with the positive or negative regulatory effects PARP-1 has on transcription of various genes [12], and also compatible with the current understanding on how Smad complexes regulate transcription, by reading the pre-existing code of local chromatin and thus providing differential gene regulation according to cell type, developmental stage and crosstalk with other signaling inputs that a given cell receives [1], [2], [3].

In conclusion, the new evidence that implicates PARP1/2 and PARG as regulators of Smad function and overall transcriptional control by the TGFβ pathway (Fig. 10c), opens a new window of understanding of the molecular connections that exist between PARP family members and the central players of a major developmental signaling pathway. Since PARG silencing blocks basic TGFβ signaling responses, development of specific PARG inhibitors may provide a potential tool that could simultaneously modulate PARG and TGFβ activity during various diseases such as cancer [21], [27]. The present investigation opens the way for exploring such novel possibilities in basic biology and in the targeted therapy of disease.

## Materials and Methods

### Cell culture and transfections

Human embryonic kidney 293T cells were cultured according to protocols from the American Type Culture Collection (LGC Standards AB, Borås, Sweden). Human immortalized keratinocytes HaCaT were obtained and cultured as described before [28]. Transient transfections of cells were done using calcium phosphate [29] and Fugene HD (Roche Diagnostics Scandinavia AB, Bromma, Sweden) according to their standard protocols. Short-interfering RNA (siRNA) oligoneucleotide pools were purchased from Dharmacon/Thermo Fischer Scientific Inc. (Waltham, MA, USA). Transfection of siRNA oligonucleotides (10–25 nM) targeting human PARP-1 (Dharmacon ONTARGETplus SMARTpool L-006656-00), human PARP-2 (Dharmacon ONTARGETplus SMARTpool L-010127-02), human PARG (Dharmacon ON-TARGETplus SMARTpool L-011488-00) or non-targeting control (Dharmacon ONTARGETplus Non-targeting pool D-001810-10), was performed using siLentfect (Bio-Rad Laboratories AB, Solna, Sweden) transfection reagent. The cells were transfected a single time for 36 or 48 h and cultured in DMEM containing 3%, 5% or 10% fetal bovine serum prior to stimulations and cell-based assays. The cells were stimulated with TGFβ and processed for RNA isolation, immunoblotting or microscopy analysis after applying PLA.

### Plasmids and other reagents

The mammalian expression vectors pCDNA3, pCDNA3-Flag-Smad2, pCDNA3-Flag-Smad3, pCDNA3-Flag-Smad4 and pDEF3-Flag-Smad2, pDEF3-Flag-Smad3, pDEF3-Flag-Smad4 have been described [29], [30]. pGEX vectors encoding GST-Smad3, GST-Smad4 and GST-Smad3ΔMH2, have been described [29], [31]. pCDNA3.1-Myc-PARP-1 encoding Myc-tagged wild-type PARP-1, was previously described [32]. The pBC-mPARP2 and the control pBC vectors were kind gifts from Valérie Schreiber [18]. The pCS2-myc-PARG and control pCS2 vectors were kind gifts from Paola Caiafa [33]. The CAGA_12_ reporter pCAGA_12_-MLP-luc, pCMV-β-gal and pEGFP-N3, have been described before [29], [30].

Recombinant mature TGFβ1 was bought from PeproTech EC Ltd. (London, UK) and Biosource Inc. (Camarillo, CA, USA). The TGFβ1 isoform was used throughout this study and is referred to as TGFβ. The β-NAD was bought from Sigma-Aldrich Sweden AB (Stockholm, Sweden), H_2_O_2_ and Coomassie brilliant blue R250 (CBB) from MERCK KGaA (Darmstadt, Germany), high purity recombinant PARP-1, PARP-2 and PARG (20,000 U/mg, 0.1 µg/ml) isolated from insect cells after baculoviral infection were bought from Axxora, LLC/ENZO Life Sciences, GmbH (Lörrach, Germany).

### Antibodies

Mouse monoclonal anti-Flag (M2 and M5) and anti-fibronectin (F3648) antibodies were from Sigma-Aldrich Sweden AB (Stockholm, Sweden); rabbit polyclonal anti-PARP2 from Active Motif (La Hulpe, Belgium); mouse monoclonal anti-PARP-1, anti-PAI-1 (plasminogen activator inhibitor 1), anti-Smad2/3 and rabbit polyclonal anti-PAR (used for PLA with mouse anti-PARP-1) from BD Pharmingen/Transduction Laboratories (BD Biosciences, Stockholm, Sweden); mouse monoclonal anti-Smad4 (B8), mouse monoclonal anti-Myc (9E10) and anti-α-tubulin from Santa Cruz Inc. (Santa Cruz, CA, USA); rabbit polyclonal anti-Smad3 from Epitomics (Burlingame, CA, USA); mouse monoclonal anti-PAR (used for PLA with rabbit anti-PARP-2 and rabbit anti-Smad3) from Axxora, LLC/ENZO Life Sciences, GmbH (Lörrach, Germany); and rabbit polyclonal anti-phospho-Smad2 was produced in house [21], [27].

### Proximity Ligation Assay

HaCaT cells were permeabilized with 0.2% Triton X-100 in PBS for 10 min at room temperature (RT) with agitation prior to double wash with 1×PBS for 5 min with agitation. The cells were incubated with Duolink II blocking solution for 1 h at RT with agitation (80 rpm), which was removed prior to adding primary antibodies. The antibodies were diluted in Duolink II antibody diluent 1∶100 and the cells were incubated overnight at 4°C, with agitation (80 rpm). The cells were washed 3×3 min with Buffer A (Duolink, Olink Bioscience, Uppsala Sweden) prior to adding secondary probes (Duolink II), diluted with Duolink II antibody diluent 1∶5. The cells were further incubated 2 h at 37°C with agitation (80 rpm), prior to 3×3 min wash with Buffer A. Duolink Ligation stock was diluted 1∶5 in double distilled water and Duolink Ligase was added to the ligation solution from the previous step at a 1∶40 dilution under vortex condition. Ligation solution was added to each sample and the slides were incubated in a pre-heated humidity chamber for 30 min at 37°C. The slides were washed with Buffer A for 2×2 min under gentle agitation and the wash solution was tapped off after the last washing. Duolink Amplification stock was diluted 1∶5 in double distilled water and Ligation solution was tapped off from the slides. Duolink Polymerase was added to the Amplification solution at a 1∶80 dilution under vortex condition. Amplification solution was added to each sample and the slides were incubated in a pre-heated humidity chamber for 90 min at 37°C and the slides were rinsed once with Buffer A. Phallodin 488 (1∶40) and Hoechst (1∶500) (both purchased from Sigma-Aldrich Sweden AB, Stockholm, Sweden), were added to phosphate buffered saline (PBS) and the slides were incubated at RT for 10 min prior to 2×10 min wash with Buffer B (Duolink II). Slides were rinsed with double distilled water and mounted with Slowfade (Invitrogen/Life Technologies-Thermo Fischer Scientific Inc., Stockholm, Sweden) mounting medium. Pictures were taken with a Zeiss AxioPlan2 epi-microscope. The DuolinkImageTool software (Olink Bioscience, Uppsala Sweden) was used for image analysis and signal quantification. Due to the antibody species specificity requirement in PLA assays, a rabbit anti-Smad3 antibody was combined with a mouse anti-PAR antibody (Fig. 1). The same rabbit anti-Smad3 antibody was combined with a mouse anti-PARP-1 antibody (Fig. 2), whereas a mouse anti-Smad2/3 antibody was combined with a rabbit anti-PARP-2 antibody (Fig. 3). The mouse anti-PARP-1 antibody was combined with the rabbit anti-PARP-2 antibody (Fig. 5), the mouse anti-PARP-1 antibody was combined with the rabbit anti-PAR antibody (Fig. 6), and the rabbit anti-PARP-2 antibody was combined with the mouse anti-PAR antibody (Fig. 6). It is therefore obvious that for some of the PLA assays it was technically impossible to compare directly the same antibodies (e.g. Smad3 against the various PARPs or PAR).

### Co-immunoprecipitation and immunoblotting assays

293T or HaCaT cells were transfected with constructs, left without transfection and/or treated as explained in the figures. Total proteins from the cells were extracted in Nonidet-P 40 (NP-40) lysis buffer (20 mM Tris-HCl [pH 8.0], 1% NP-40, 150 mM NaCl, 2 mM EDTA, and complete protease inhibitor cocktail from Roche Diagnostics Scandinavia AB, Bromma, Sweden) and subjected to SDS-PAGE and analyzed by immunoblotting, as described previously [28]. Lysates were heated at 95°C for 5 min prior to SDS-PAGE. Alternatively, cells were lysed in the above NP-40 lysis buffer 36–48 h after transfection or after the indicated times of TGFβ stimulation. The indicated proteins were immunoprecipitated, and after three washes in lysis buffer, including one wash in lysis buffer containing 0.5 M NaCl, the immunocomplexes were resolved by SDS-PAGE and immunoblotted with antibodies, as described in the figure legends.

### In vitro ADP-ribosylation assays

Newly prepared GST-vector or GST-Smad proteins were kept on glutathione beads and incubated in 100 µl PARP-1 reaction buffer (100 mM Tris-HCl [pH 8], 10 mM MgCl_2_, 1 mM dithiothreitol), with or without 100 ng PARP-1 or 100 ng PARP-2. Then, 80 nM β-NAD and 20 nM ^32^P-β-NAD were added and the samples were incubated for 30 min at 37°C while shaking. For reactions with excess cold NAD, instead of 80 nM β-NAD, 180, 480 or 980 nM β-NAD were included in separate reactions, reaching the total concentration of cold plus radioactive β-NAD to 200, 500 and 1,000 nM respectively (Fig. S1). PARG incubations were performed in PARG reaction buffer containing (100 mM Tris-HCl, pH 8.0, 10 mM MgCl_2_ and 1 mM dithiothreitol) with and without PARG. At the end of each reaction, beads with GST fusion proteins were collected via centrifugation, followed by a quick double wash in ice-cold NP-40 lysis buffer to remove excess radioactive β-NAD. Samples were then heated for 4 min at 95°C in sample buffer and subjected to SDS-PAGE. Gels were fixed, stained with CBB and dried before measuring radioactivity in a Fuji-X Bio-Imager (FujiFilm Corp., Stockholm, Sweden).

### Luciferase Assays

HaCaT cells were transiently transfected with TGFβ/Smad-responsive promoter-reporter pCAGA_12_-MLP-luc for 36–48 h prior to stimulation with TGFβ. pCMV-β-gal or pEGFP were co-transfected as controls for normalization. Additional constructs or siRNAs were included in the transfections according to the figures. Luciferase reporter assays were performed with the enhanced luciferase assay kit from BD PharMingen, Inc. (BD Biosciences, Stockholm, Sweden), according to the protocol of the manufacturer. Normalized promoter activity data are plotted in bar graphs that represent average values from triplicate determinations with standard deviations. Each independent experiment was repeated at least twice.

### Real-time RT PCR

HaCaT cells were treated as indicated in figures before extraction of RNA using RNeasy (Qiagen AB, Solentuna, Sweden). Measurements of mRNA expression were performed as described [30]. The primers used for PCR amplification were: human *PARP-1*, forward, 5′-AAGCCCTAAAGGCTCAGAAC G-3′, reverse, 5′-ACCATGCCATCAGCTACTCGGT-3′; human *PARP-2*, forward, 5′-GGTCATGGGCCAGCAAAAGGG-3′, reverse, 5′-CATGAGCCTTCCCCACCTTGG-3′; human *PARG*, forward, 5′-GAAAGGGACGACTGGCAGCGG-3′, reverse, 5′-CCAAAGGCACCACAGCCCCA-3′; human *GAPDH*, forward, 5′-GGAGTCAACGGATTTGGTCGTA-3′, reverse, 5′-GGCAACAATATCCACTTTACC A-3′; human *Fibronectin*, forward, 5′-CATCGAGCGGATCTGGCCCC-3′, reverse, 5′-GCAGCTGACTCCGTTGCCCA-3′; human *SMAD7*, forward, 5′-ACCCGATGGATTTTCTCAAACC-3′, reverse, 5′-GCCAGATAATTCGTTCCCCCT-3′; human PAI-1, forward 5′-GAGACAGGCAGCTCGGATTC-3′, reverse, 5′-GGCCTCCCAAAGTGCATTAC-3′.

### Statistical analysis

The differences between mRNA levels under control, gene specific silencing and protein over-expression conditions were evaluated statistically using a standard two-tailed t-test for samples with unequal variance and two-sample with equal variance, respectively. Significance is reported at *p*<0.05.

## Supporting Information

Figure S1 **GST-Smad proteins used for in vitro ADP-ribosylation assays.**
(EPS)

Figure S2 **Competition of Smad3 ADP-ribosylation by cold β-NAD.**
(EPS)

Figure S3 **Endogenous PARG depletion suppressed fibronectin and PAI-1 protein induction by TGFβ1.**
(EPS)
